# Black Queens of Fruits: Chemical Composition of Blackberry (*Rubus* subg. *rubus* Watson) and Black Currant (*Ribes nigrum* L.) Cultivars Selected in Serbia

**DOI:** 10.3390/foods12142775

**Published:** 2023-07-21

**Authors:** Zaklina Karaklajic-Stajic, Jelena Tomic, Marijana Pesakovic, Svetlana M. Paunovic, Franci Stampar, Maja Mikulic-Petkovsek, Mariana C. Grohar, Metka Hudina, Jerneja Jakopic

**Affiliations:** 1 Department for Technology of Fruit Growing, Fruit Research Institute, Kralja Petra I No 9, 32 000 Cacak, Serbia; zaklinaks@yahoo.com (Z.K.-S.); jtomic@institut-cacak.org (J.T.); mpesakovic@institut-cacak.org (M.P.); svetlana23869@gmail.com (S.M.P.); 2 Department of Agronomy, Biotechnical Faculty, University of Ljubljana, Jamnikarjeva 101, SI-1000 Ljubljana, Slovenia; franci.stampar@bf.uni-lj.si (F.S.); maja.mikulic-petkovsek@bf.uni-lj.si (M.M.-P.); marianacecilia.grohar@bf.uni-lj.si (M.C.G.); metka.hudina@bf.uni-lj.si (M.H.)

**Keywords:** berries, sugars, organic acids, phenolic compounds, frozen storage

## Abstract

Black fruits, especially blackberries and black currants, are highly appreciated by consumers due to their nutraceutical properties, which have reported health benefits. This study aimed to assess the fruit quality of the blackberry (cv. ‘Čačanska Bestrna’) and black currant (cv. ‘Čačanska Crna’) created at the Fruit Research Institute, Čačak (Republic of Serbia) by evaluating basic quality parameters (fruit weight, soluble solids), and content of primary (sugars and organic acids) and secondary (phenolic compounds) metabolites. Additionally, the study examined the quality of frozen and long-term frozen storage (6, 9, and 12 months). The results showed that ‘Čačanska Crna’ contained a high level of total soluble solids (15.23°Bx), sugars (93.06 mg g^−1^), and a high index of sweetness (159.48) as well as a high content of acids (34.35 mg g^−1^) in the fruit. Both species were found to contain forty-seven phenolic compounds, including phenolic acids, flavanols, and anthocyanins, analyzed using high performance liquid chromatography-mass spectrometry (HPLC-MS). ‘Čačanska Bestrna’ had the highest amounts of phenolic acids (66.85 mg 100 g^−1^) and flavanols (53.99 mg 100 g^−1^), whereas ‘Čačanska Crna’ showed the highest levels of flavonols (8.57 mg 100 g^−1^) and anthocyanins (139.11 mg 100 g^−1^). Furthermore, the study revealed that anthocyanins were the most abundant phenolic group in both blackberries and black currants, and their levels remained constant during frozen storage. The sugar content in both species remained unchanged, while the acid concentration increased over time in blackberries but remained consistent in black currants. Overall, the findings highlight the superior phenolic content, particularly anthocyanins, of the cultivars with black fruits selected in Serbia. These cultivars have great commercial potential for the fresh market and processing. Moreover, the study suggests that frozen storage is an effective method for preserving their quality and beneficial properties.

## 1. Introduction

Berry fruits are often the richest source of phenols among fruits and vegetables [1] and attract a lot of attention due to their positive role in human health and the prevention of diseases. Among them, black fruits, such as blackberries and black currants, are good sources of natural antioxidants and have long been recognized as being especially rich in many compounds that have a high potential to benefit human health [2].

In blackberries, the high content of phenols, especially anthocyanins and ellagitannins, as well as other phenolic compounds, contribute to their high antioxidant capacity [3]. In addition, blackberries are also rich in dietary fiber, vitamins, and minerals [4]. On the other hand, blackcurrants also have significant antioxidant activity, partly due to their relatively high content of ascorbic acid [5] and phenolic compounds, anthocyanins and hydroxycinnamic acids being the most important among them [6].

Among the 43 cultivars of different fruit species that were developed during seventy years of work on fruit breeding at the Fruit Research Institute in Čačak (Serbia), within the berry species, the blackberry ‘Čačanska Bestrna’ and the black currant ‘Čačanska Crna’ are commercially the most important.

In the past decade, according to Strik et al. [7], Serbia was among four world leading blackberry producers with a 69% share of total European and 18% of total word production, ranking fourth in world blackberry production after USA, Mexico, and China. The same authors point out that in the world of commercial blackberry plantings, the most represented cultivars from the group of semi-erect growth are ‘Thornfree’, ‘Loch Ness’, and ‘Chester Thornless’, representing about 58%, followed by ‘Dirksen Thornless’, ‘Hull Thornless’, and ‘Smoothstem’ with about 28% and ‘Čačanska Bestrna’ with about 5%. Due to good adaptability to different agroecological conditions, ‘Čačanska Bestrna’ is dominant in blackberry plantings in Serbia, accounting for 60% of the total surface, despite a significant number of other introduced cultivars. Apart from good adaptability, this cultivar has high yields and large fruits of exceptional nutritional value, which overall represents the most important goals of blackberry breeding programs [8]. Stanisavljević et al. [9] emphasized that this cultivar has exceptional potential in terms of yield, which is a key element in the profitability of blackberry production.

The *Ribes* genus is important in world berry production, especially as currants rank second in consumer preferences immediately after strawberries [10]. Although they are grown all over the world in cooler climates, most of the commercial currant production occurs in northern Europe. In the *Ribes* genus, the most important species are black currant (*Ribes nigrum* L.) and red currant (*Ribes rubrum* L.) [11]. The production of black currant in Serbia is insufficient, although there are optimal agro-environmental conditions for its growth. Among black currant plantings, ‘Čačanska Crna’ is the most frequent cultivar. Its berries are characterized by exceptional potential with regard to the profile and content of phenolic compounds, especially anthocyanins, which affect fruit color, taste, and medicinal properties [12]. This cultivar was also used as a parent in many current breeding programs in the world.

Fruits of blackberry and black currant produced in Serbia are mostly placed in the domestic and international markets in a frozen state because the processing facilities are scarce. Freezing is generally considered to be the least destructive preservation technology for phenolic compounds in berries and is recommended as a pretreatment for manufacturing other berry products, although the physical and nutritional quality of frozen fruit can somehow be affected by the freezing method, packaging material, storage conditions, cultivar, and maturity stage [13]. In modern food technology, the trend is to maximize nutrient retention during both storage and processing [14]. However, the literature data related to the effects of long-term frozen storage on the chemical composition of berries are still scarce and require further research.

The previous study of cultivars ‘Čačanska bestrna’ and ‘Čačanska Crna’ were mainly related to the examination of growth characteristics, yield, and basic physiochemical attributes of fruits [9,15,16]. The only available results are those of Mikulic-Petkovsek et al. [1,17,18] who studied the chemical composition of blackberry cultivars at different stages of maturity and determined that ‘Čačanska bestrna’ had the largest fruit with the highest vitamin C content compared to the other studied blackberry cultivars. Also, a comparative study of the pomological characteristics of 11 black currant cultivars showed the superiority of ‘Čačanska Crna’ in terms of vigor, berry size, and suitability for mechanized harvesting [19]. However, there is no detailed report on the biochemical composition of these two cultivars.

Metabolome assays add a new dimension to these studies, since they focus on the biochemical contents of cells and tissues and have a rapidly growing significance in the knowledge of fruits’ role in human health. Therefore, in addition to the assessment of fruit quality and main yield parameters, the goal of this study was to investigate the effect of freezing and long-term frozen storage (6, 9, 12 months) on the nutritional composition of black fruit (blackberry cv. ‘Čačanska Bestrna’ and black currant cv. ‘Čačanska Crna’) originating from the breeding program of Fruit Research, Čačak (Serbia). We hypothesize that the good yield and external characteristics of the fruits of these two berry species will also be supported by the high internal quality of the fruit, which is reflected in the content of selected primary and secondary metabolites after freezing and long-term storage. The results obtained from this study will help producers expand the production of these varieties in areas with similar agroecological conditions and indicate to the food industry the stability of plant metabolites during storage.

## 2. Materials and Methods

### 2.1. Plant Material and Experimental Design

Blackberries and black currants were planted in 2008 at the experimental station of the Fruit Research Institute, located in Čačak (43°54′ N latitude, 20°22′ E longitude, 225 m altitude), Western Serbia. Blackberries were planted at a spacing of 3.0 m in the row and 1.5 m within the row and trained to a three-wire trellis. Black currants were planted at the same row spacing in the bush training system. Fertilization, weed control, and irrigation standards for the region were provided during the growing seasons.

The study involved the blackberry cultivar ‘Čačanska Bestrna’ and black currant cultivar ‘Čačanska Crna’, both developed under the Fruit Research Institute, Čačak breeding program (Republic of Serbia) (Table 1).

The experiment was set up as a randomized block design with five bushes per plot replicated four times (20 bushes in total) for each genotype. Fully ripe berries were harvested in the middle of June for black currants and at the beginning of August for blackberries in 2018. Fully ripe (optimally ripe) blackberry fruits were black, glossy, and easy to pick from branches [20], while fruits of black currant have been picked when all the berries of the cluster became fully black [1]. In order to obtain uniform samples berries were visually chosen from the same pool of each bush at the same development stage the fruits were hand-picked.

Samples of both studied species (blackberry and black currant) were taken in four replicates, and each replicate contained 0.5 kg of fruit. The samples were taken to the laboratory and frozen in liquid nitrogen and randomly divided into four groups: (1) fruits stored for several days until chemical analyses; (2) 6 months; (3) 9 months; and (4) 12 months of frozen storage at −20 °C. Firstly, a detailed chemical analysis was performed on fruits that were stored in a frozen state for several days. Samples that were stored in long-term frozen conditions (6, 9, and 12 months) were analyzed immediately afterward. Bearing in mind that total sugars, organic acids, and anthocyanins in berries have a strong influence on the sensory quality and thus influence the acceptance of berries and berry products by consumers [21], analyses of the mentioned parameters were performed on long-term frozen fruits.

### 2.2. Meteorological Data and Soil Characteristics

**Figure 1:**
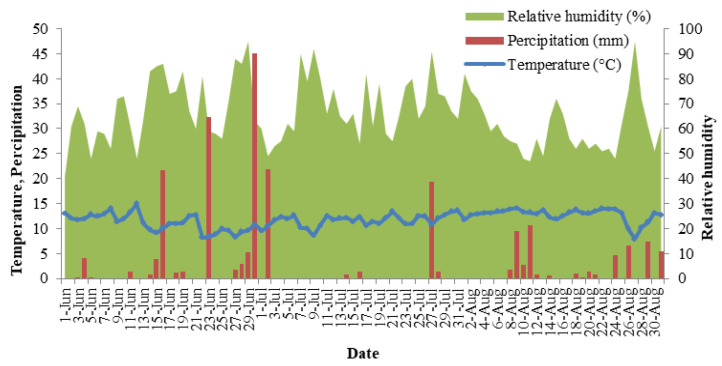
Variation of temperature, precipitation, and relative humidity from 1 June to 30 August 2018.

Climate variables, including temperature, precipitation, and relative humidity were recorded from 1 June to 30 August 2018. Data were obtained from the Republic Hydrometeorological Service of Serbia (Figure 1). The soil in the blackberry planting was vertisol, moderately supplied with organic matter (2.92%); soil pH in KCl 0.01 mol L^−1^ was 4.98. The contents of available soil P and K were 4.64 and 29.23 mg 100 g^−1^, respectively.

### 2.3. Determination of Productivity and Fruit Quality Parameters

After harvest, 25 fruits in each replication (n = 4) were randomly selected to determine average fruit weight using a technical balance (PB3002-S, Mettler Toledo, Greifensee, Switzerland) with an accuracy of ±0.01 g, and the data were expressed in g. The total soluble solids content (TSS) was determined by a digital refractometer (Carl Zeiss, Jena, Germany) at 20 °C, and data were expressed in °Bx. Yield per bush was assessed by measuring the weight of all picked fruits from each harvest on an electronic scale (ACS-A, Gromy Scale Co., Ltd., Hong Kong, China), and presented as kg bushes^−1^. Yield per unit area was calculated by multiplying the yield per bush with the number of plants per ha (2.222 plants ha^−1^ for both blackberry and black currant) and values were expressed in tons per hectare (t ha^−1^).

### 2.4. Determination of Sugars and Organic Acids

Berries of both cultivars (2.0 g) were homogenized in 10 mL of double-distilled water. After 30 min at 10,000 rpm on the centrifuge (5810 R, Eppendorf, Hamburg, Germany), they were filtered through a 0.20 µm cellulose ester filter (Macherey-Nagel, Dueren, Germany) and poured into a vial before being analyzed by a high-performance liquid chromatography system (HPLC, Thermo Scientific, Finnigan Spectra System, Waltham, MA, USA), as described by Jakopic et al. [22]. The separation of sugars was performed using a Rezex RCM-monosaccharide Ca^2+^ (8%) column (Phenomenex, Torrance, CA, USA) coupled with a Chromsep guard column operated at 65 °C (300 mm × 7.8 mm) and detected with a refractive index (RI) detector. The mobile phase was double-distilled water, the flow rate was 0.6 mL min^−1^, and the run time was 30 min. Organic acids were analyzed using the same HPLC system, equipped with a UV detector set at 210 nm, and a Rezex ROA-Organic Acid H+ (8%) column (Phenomenex, Torrance, CA, USA) coupled with a Chromsep guard column, as described by Mikulic-Petkovsek et al. [17]. The column temperature was set at 65 °C. The elution solvent was 4 mM sulfuric acid at a flow rate of 0.6 mL min^−1^. The duration of the analysis was 30 min. The contents of sugars and organic acids were calculated according to a calibration curve of corresponding standard solutions of external standards. The results were expressed as g per kg of fresh weight for sugars and organic acids (g kg^−1^). The sweetness was calculated to determine the sweetness perception of berries, based on the fact that fructose and sucrose are 2.30 and 1.35 times, respectively, sweeter than glucose, using the equation:

Sweetness Index = (1.00 × [glucose]) + (2.30 × [fructose]) + (1.35 × [sucrose])(1)

The sugar/organic acid ratio and maturity index were calculated following equations:

Sugar/organic acid ratio = total sugars content/total acid(2)

Maturity index = total soluble solids/total acid(3)

### 2.5. Determination of Phenolic Compounds

For the analysis of individual phenolic compounds, berries were chopped and 1.0 g of the sample was extracted with 5 mL of methanol with 5% formic acid in a cooled ultrasonic bath for 1 h. Supernatants were centrifuged for 10 min at 10,000 rpm, filtered through 0.2 µm polyamide filters (Macherey-Nagel, Dueren, Germany), and transferred into a vial injected into the HPLC system coupled with a diode array detector-DAD (Thermo Finnigan, San Jose, CA, USA) at 280 nm, 350 nm, and 530 nm, as described by Jakopic et al. [22]. The column was a Gemini C18 (150 × 4.4 mm, 3 µm; Phenomenex, Torrance, CA, USA) coupled with a Chromsep guard column operated at 25° C. The elution solvents were aqueous 0.1% formic acid (Fluka Chemie GmBH, Buchs, Switzerland) in double-distilled water (Milli-Q system, Millipore, Bedford, USA) with 3% acetonitrile (Fluka Chemie GmBH, Buchs, Switzerland) (A) and 0.1% formic acid in acetonitrile with 3% double-distilled water (B). Samples were eluted using a linear gradient from 5 to 20% B in the first 15 min, followed by a linear gradient from 20 to 30% B for 5 min, an isocratic mixture for 5 min, a linear gradient from 30 to 90% B for 5 min, and an isocratic mixture for 15 min before returning to the initial conditions.

Phenolic compounds were identified using a mass spectrometer (LTQ XL Linear Ion Trap Mass Spectrometer, Thermo Fisher Scientific, Waltham, MA, USA) with electrospray ionization (ESI) operating in a positive (anthocyanins) or negative (all the other phenolics) ionization mode. The analysis was performed using full scan, data-dependent MSn scanning from *m*/*z* 100 to 1700. All of the mass spectrometer conditions set were the same as reported by Mikulic-Petkovsek et al. [17]. Spectral data were elaborated using the Excalibur software 4.1.50 software (Thermo Fisher Scientific, Waltham, MA, USA). The identification of compounds was confirmed by comparing retention times and their spectra, by adding the standard solution to the sample, and by fragmentation and comparison to the literature data.

The content of individual phenolic compounds was calculated on the basis of peak areas of the samples and the corresponding standard compounds, and obtained values were expressed in mg per 100 g fresh weight (mg 100 g^−1^). The content of phenols of which standard compounds were not accessible, was given using similar compounds: 3-*p*-coumaroylquinic acid and *p*-coumaric acid hexosides as *p*-coumaric acid, caffeic acid hexosides as caffeic acid, ferulic acid hexoside as ferulic acid, procyanidin dimer, and trimers as procyanidin B1, quercetin derivatives as quercetin-3-glucoside, kaempferol derivatives as kaempferol-3-glucoside, petunidin-3-rutinoside as petunidin (Fluka Chemie GmBH, Buchs, Switzerland); ellagic acid derivatives as ellagic acid, cyanidin derivatives as cyanidin-3-glucoside, pelargonidin derivatives as pelargonidin-3-glucoside delphinidin derivatives as delphinidin-3-glucoside (Sigma-Aldrich, Steinheim, Germany); myricetin derivatives as myricetin-3-rhamnoside (Apin Chemicals LTD, Abingdon, UK). 

### 2.6. Determinations of Sugars, Organic Acids, and Anthocyanins Content during Long-Term Frozen Storage

Individual invert sugars (glucose, fructose, and sucrose) were determined by HPLC (Waters Breeze, Milford, CT, USA) using a procedure described by Milivojević et al. [23]. The detection of sugars was performed by a 2465 Waters electrochemical detector (Waters Breeze, Milford, CT, USA). The column was CarboPac PA1 (Dionex, Sunnyvale, CA, USA). Sugars were eluted with 200 mmol L^−1^ NaOH for 20 min, at 1.0 mL min^−1^ flow rate at 30 °C constant temperature. The absorbance was measured in the range of 210–327 nm. Organic acids (citric acid, malic acid) were analyzed using a Hewlett-Packard HP1100 system equipped with a photodiode array detector (Palo, Alto, CA, USA). The column was Aminex—HPX-87H (Bio-Rad Laboratories, Hercules, CA, USA) operated at 40 °C. The absorbance was measured in the range 490–600 nm.

Quantification of anthocyanins was performed by reversed-phase HPLC, using the modified method of Mišan et al. [24]. Analysis was performed using HPLC (Agilent 1200 series, Santa Clara, CA, USA), equipped with DAD and Chemstation B.04.03-SR2 Software (Agilent Technologies, Santa Clara, CA, USA), a binary pump, vacuum degasser, an autosampler and a thermostatted column compartment (Zorbax Eclipse Plus-C18, 1.8 μm, 600 bar, 2.1 × 50 mm, Agilent, Santa Clara, CA, USA), at a flow rate of 0.8 mL min^−1^. Gradient elution was performed by varying the proportion of solvent A (methanol) to solvent B (1% formic acid in water (*v/v*)) as follows: initial 0–2 min, 100% B; 2–4 min, 100–98% B; 4–6 min, 98–95% B; 6–7 min, 95–73% B; 7–10 min, 75–48% B; 10–12 min 48% B; 12–20 min, 48–40% B. The total running time and post-running time were 21 and 5 min, respectively. The column temperature was 30 °C. The injection volume for samples and standards was 5 μL using an autosampler.

The content of all sugars, organic acids, and anthocyanins, determined during long-term frozen storage (6, 9, 12 months), were summed and presented as the sum of sugars, acids (g kg^−1^) and anthocyanins (mg 100 g^−1^) of fresh weight. The sugars/acid ratio is defined as the proportion of total sugars and total acids in the samples.

### 2.7. Statistical Analysis

The data were presented as mean ± standard deviation. Differences between means were compared by the Student’s test using the data analysis software system Statistica version 8.0 (Statsoft. Inc., Tulsa, OK, USA). The significance of differences at a 5% level among means was determined using the independent samples *t*-test procedure.

Fisher LSD (Least Significant Difference) test was performed to test any significant differences among periods of frozen storage (6, 9, 12 months) considering *p* ≤ 0.05 as statistically significant for all analyses performed.

## 3. Results and Discussion

### 3.1. Productivity and Basic Fruit Quality Parameters

Fruit quality can be defined by weight and soluble solids content, while yield is considered the most important parameter of production efficiency. These are the main factors that have a direct effect on marketability [25]. Among fruit physical properties, fruit weight (size) is a major quantitative inherited factor determining yield, fruit quality, and consumer acceptability in both fresh markets and the processing industry [26]. In blackberries, large fruits are preferred by consumers, but excessive berry weight (possibly > 15.0 g) is usually not desired for processed or fresh market use [25]. The fruit weight of blackberries in our study (Table 2) was in the optimal range for ‘Čačanska Bestrna’, and the obtained results were higher than the findings of Milivojević [27] and Milošević et al. [15].

Black currant fruits are larger (average weight 0.8–2 g) compared to red and white currant fruits (average weight 0.4–1 g). According to the average weight, black currant cultivar ‘Čačanska Crna’ (Table 2) belongs to the group of cultivars with small fruits (fruit weight up to 1 g) [28]. Similar data were reported by Stanisavljević et al. [29], Đorđević et al. [28], and Milivojević et al. [11], which was also confirmed in our study, where the average weight of black currant cultivar ‘Čačanska Crna’ was 1.05 g.

The TSS is a good indicator of maturity in blackberry and black currant and is also very important in the food industry [8]. The TSS is closely associated with fruit sweetness, and consumers tend to select fruits that taste sweeter. In our study, the average TSS of black currant ‘Čačanska Crna’ was 15.23 °Bx, which was higher than the data reported for this cultivar before [2,28,29], while blackberry ‘Čačanska Bestrna’ measured 9.40 °Bx, which was in agreement with a previous study [30]. Also, it is important to note that ‘Čačanska Bestrna’ showed a higher value of TSS compared to its parental cultivars (‘Dirksen Thornless’ and ‘Black Satin’) [25]. The aforementioned results of TSS in the blackberry ‘Čačanska Bestrna’ and black currant ‘Čačanska Crna’ makes them suitable for fresh consumption and raw material for industrial processing into juices, jams, jellies, puree, liquors, and extracts for nutritional supplements [31].

Fruit yield is determined by the interaction of growing conditions, physiological properties, and morphological traits [32]. Our results for the average yield per bush and per unit area obtained for ‘Čačanska Bestrna’ (8.01 kg and 17.80 t ha^−1^) were confirmed by Stanisavljević et al. [9]. In addiion, this blackberry cultivar is superior to its parental cultivar in fruit weight and yield per bush [9,25]. In black currant ‘Čačanska Crna’, yield per bush was comparable to the values for the same cultivar reported before [28]. According to the level of productivity, this cultivar is classified as a medium yielding. Despite the fact that the black currant ‘Čačanska Crna’ is a medium-yielding cultivar, its resistance to frosts and sunburns [19] represents a great potential for expansion to areas with various agro-ecological conditions.

### 3.2. Primary Metabolites: Sugars and Organic Acids

Sensory attributes and nutritive value of fruits play an important role in consumer satisfaction and influence their further consumption. Sweetness is mostly attributable to mono- and disaccharides rather than to other compounds. The sour taste is reliably linked to organic acids and pH. The compounds responsible for taste are mostly water-soluble and non-volatile (carbohydrates, organic acids), in contrast to compounds responsible for aroma, which are characterized by instability [33]. Sourness induced by various organic acids in berries can have a negative influence on the pleasantness, but it can also be a positive feature to some extent [21]. Organic acids are useful for the stabilization of ascorbic acid and anthocyanins, which is why they play a key role in the formation of fruit color and the extension of storage capacity of fresh and processed fruits [34]. In addition, acids are also important in fruit processing because they affect the gelation properties of pectin [35]. Five organic acids were determined in the studied berry cultivars: citric, malic, quinic, shikimic, and fumaric acids (Table 3). In both black-colored fruits, citric acid was the dominant organic acid, accounting for 41% (‘Čačanska Bestrna’) and 70% (‘Čačanska Crna’) of the total organic acids content. The level of citric acid in our study for ‘Čačanska Bestrna’ was higher than in study of the Mikulic-Petkovsek et al. [1] and comparable to the results of Veberic et al. [36]. On the other hand, the fruits of black currant ‘Čačanska Crna’ accumulated higher levels of citric acid than those obtained by Milivojević et al. [11]. The level of malic acid in our samples was low, which is consistent with Mikulic-Petkovsek et al. [17], who reported that sweet fruits do not necessarily have high sugar content, but do generally contain characteristically low levels of organic acids, especially malic acid. Sweetness is usually correlated with citric acid, shikimic acid, and sugar/organic acid ratio [37], which is also confirmed by our results in the black currant cultivar ‘Čačanska Crna’.

Three different sugars (glucose, fructose, and sucrose) were determined in both cultivars ‘Čačanska Bestrna’ and ‘Čačanska Crna’ (Table 3), and these results were similar to previous results [10,13]. Sugar amounts have been reported as comparatively lower in blackberries than in any other berry fruits [16]. Furthermore, fruit with identical total sugar content but relatively more fructose or sucrose taste sweeter than those with higher glucose content [34]. In our study, more than half of the total sugars content in black currant (93.06 mg g^−1^ FW) was fructose (49.95 mg g^−1^). Overall, the determination of sugar content in berries is very important for sweetness perception and consumer acceptance of fruits [35], which makes its identification and quantification crucial.

The sugar/organic acid ratio of optimally mature blackberries and black currants in our study were 3.39 and 2.77, respectively. This value is one of the key quality indexes for blackberries and currants [38], determining their taste and the final quality of the derived products [39].

### 3.3. Secondary Metabolites: Phenolic Compounds

Phenolic compounds are among the most relevant bioactive compounds regarding the nutraceutical value of berries, and their profile and content also influence the quality and flavor of the fruit. They comprise a large group of secondary metabolites with one or more aromatic rings and variable degrees of hydroxylation, methoxylation, and glycosylation, which contribute to fruit color, astringency, and bitterness [39]. The groups of phenolic compounds in berries are phenolic acids, flavonols, and anthocyanins [38].

A total of 47 phenolic compounds were determined in the analyzed blackberry and black currant cultivars, including 13 phenolic acids (hydroxycinnamic and hydroxybenzoic acids), 6 flavanols, 17 flavonols, and 11 anthocyanins (Table 4).

#### 3.3.1. Phenolic Acids

Phenolic acids like chlorogenic acid and neochlorogenic acid exhibit antioxidative properties by chelating metal ions, inhibiting lipid oxidation, inhibiting radical-forming enzymes, and eliminating free radicals [40]. However, the role of phenolic compounds in human health is still not clear, since different studies show that they can have both oxidative and antioxidative behavior depending on the metabolic context [41,42].

A significant share of the total phenolic content in blackberry fruit consisted of hydroxycinnamic acids. We identified seven different phenolic acids in blackberry fruits, among which neochlorogenic acid is predominant (59.28 mg 100 g^−1^). In addition to caffeic acid hexosides 1 and 2 (1.48 and 3.30 mg 100 g^−1^, respectively), the sum of all the rest of the phenolic acids was lower than 1 mg 100 g^−1^. Contrary to our results, Mikulic-Petkovsek et al. [17] observed a lower value of hydroxycinnamic acids in cv. ‘Čačanska Bestrna’ at different ripening stages. In contrast, Poledica et al. [43] measured a 30-times lower content of hydroxycinnamic acid derivatives in the red fruits of raspberry ‘Willamette’, the most common raspberry cultivar grown in Serbia. In general, raspberries contain an average of 70–90 mg 100 g^−1^ of total phenolic acids, which is comparable to the content in the blackberry cultivar ‘Čačanska Bestrna’.

On the other hand, black currant ‘Čačanska Crna’ contained only ferulic acid (0.27 mg 100 g^−1^) and caffeic acid hexoside (6.21 mg 100 g^−1^), similar to previous report [44], which recorded that main phenolic acid in black currant was caffeic acid. Ferulic acid (FA) is an abundant dietary antioxidant that may offer beneficial effects against cancer, cardiovascular disease, diabetes, and Alzheimer’s disease [45]. However, the levels of ferulic (0.71 mg 100 g^−1^), and caffeic acids (2.17 mg 100 g^−1^) detected by Gavrilova et al. [46] in black currant in Macedonia were not coincident with our results.

Blackberries contained fewer hydroxybenzoic acids than raspberries, in which they are dominant and mostly present in the form of ellagic acid derivatives [40]. Particularly, ellagic acid is a hydroxybenzoic acid that is found in blackberries and black currants among ellagitannins, which show diverse biological activities with positive effects on human health [47]. The results from recent research have increased the interest in ellagic acid, both as a potential protective agent of the liver and skin and as a potential anticancer agent, due to the specific mechanisms affecting cell proliferation, apoptosis, DNA damage, and angiogenesis and its aforementioned anti-inflammatory properties [48]. These compounds have been found in the genus *Rubus* (raspberry, blackberry, cloudberry, and arctic bramble), *Fragaria* (strawberry), and other fruit species (pomegranates, grapes, cranberry, blueberry, guava, walnuts, and some other nuts) [49]. The free form of ellagic acid obtained after hydrolysis in different berries varied from 2 to 34 mg 100 g^−1^ [46,47]. Values of ellagic acid derivatives in our study were in range with previously reported data on blackberries [47,48], but significantly higher compared to Mikulic-Petkovsek et al. [18]. Contrary to blackberry, black currant contained only ellagic acid deoxyhexoside. Overall, hydroxycinnamic acids had higher content than hydroxybenzoic acids in both examined cultivars.

#### 3.3.2. Flavanols

Berries or certain parts of the berries may be especially bitter and astringent due to their phenolic compounds [50]. Among them, flavanols, besides adding a characteristic astringency to berries, also have positive effects on metabolic disorders (diabetes, obesity, and metabolic syndrome) [51]. Flavanols were the second most abundant group among phenolic compounds in the blackberry cultivar ‘Čačanska Bestrna’ after anthocyanins. In blackberry fruits, catechin, epicatechin, and their polymerized forms are the most frequent flavanols [52]. Our results (Table 4) coincide with these findings, considering that they were also found in the studies cultivars, together with 4 procyanidins that have been identified in blackberry. Interestingly, in contrast to blackberry, in the fruit of black currant, no flavanols were found. Previous research suggests that the metabolic pattern of flavanols during fruit ripening is species- and cultivar-dependent [1,18]. Of all the analyzed phenols in blackberry, flavanols accounted for 24%, among which procyanidins were predominant with 71%. In the fruit of ‘Čačanska Bestrna’, a higher diversity of flavanols was detected in our samples compared to the results Mikulic-Petkovsek et al. [1], which analyzed the same cultivar in a different geographical location. These results suggest that cultivars exhibit their full biopotential in the region of origin.

#### 3.3.3. Flavonols

**Figure 2:**
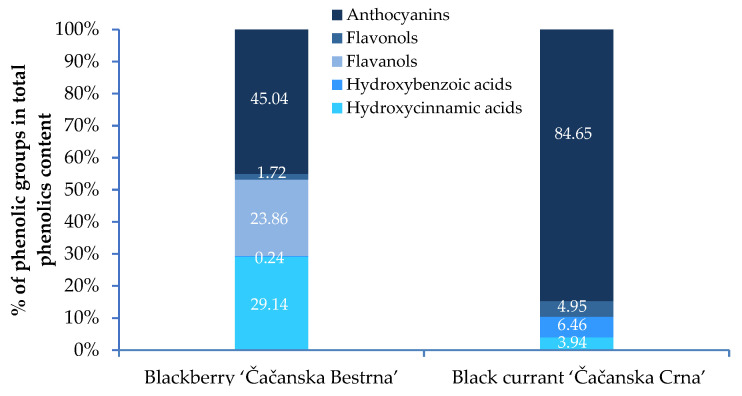
Proportion of different phenolic groups in berry cultivars selected in Serbia.

In the studied blackberry cultivar, flavonols were less abundant than phenolic acids, flavanols, and anthocyanins. Nine different quercetin derivatives, isorhamnetin-3-glucuronide, and kaempferol glycosides were identified in the fruit of ‘Čačanska Bestrna’. Quercetin-3-galactoside predominated, while the other identified flavonols were present in smaller amounts (<0.60 mg 100 g^−1^). Mikulic-Petkovsek et al. [17] measured lower levels of flavonols in five blackberry cultivars during three harvest times. In the black currant cultivar ‘Čačanska Crna’, four quercetin, three myricetin, and two kaempferol derivatives were quantified. Myricetin was the major flavonol, followed by quercetin and kaempferol, which is similar to the study of Gavrilova et al. [46]. The total proportion of flavonols in total phenolics content ranged from 1.7 (blackberry) to 4.9% (black currants) (Figure 2). Flavonols are a highly desirable group of secondary metabolites receiving increased attention, mainly due to their beneficial pharmaceutical properties, such as anticancerogenic effects [37]. The content of flavonols in plums and raspberries, the most economically important fruit species in Serbia, is lower compared to the examined blackberry and blackcurrant cultivars. Further, Sultana et al. [53] pointed out that the content of flavonols varies widely, ranging from the highest values in mulberry to the lowest in plum.

#### 3.3.4. Anthocyanins

Anthocyanins are subgroups of natural flavonoids and are associated with fruit coloration [34]. The content and profile of anthocyanins in berry fruits are determined by genotype and different environmental factors [2,33,51]. In our study, higher total anthocyanin content was determined in black currant (139.11 mg 100 g^−1^) compared to ten cultivars grown in America [52]. Similar amounts of anthocyanins were detected in blackberries (101.93 mg 100 g^−1^). Generally, anthocyanins contributed the most to the total phenolic content (Figure 2), in both blackberries and black currants (45% and 85%, respectively).

Eight anthocyanins were identified in the blackberry cultivar ‘Čačanska Bestrna’ (Table 4), of which cyanidin-3-glucoside was the most common, accounting for more than 80% of total analyzed anthocyanins. A similar presence of cyanidin-3-glucoside in blackberries was also described by Kolniak-Ostek et al. [54] and Mikulic-Petkovsek et al. [18]. Five different anthocyanins were determined in samples of black currant cultivar ‘Čačanska Crna’. Delphinidin-3-rutinoside was the prevailing anthocyanin (42.53%) while cyanidin-3-rutinoside and delphinidin-3-glucoside accounted for approximately 44.5% of all analyzed anthocyanins. The level of total anthocyanins in our samples was similar to the data of Gavrilova et al. [46], while Karaagac and Sahan [55] reported even higher amounts of anthocyanins in black currant. The role of anthocyanins in human health has been a focus before. Particularly, anti-inflammatory effects in both in vitro and in vivo models have been reported in anthocyanins from berries [56], as well as their antioxidant, anticarcinogenic, and neuroprotective properties. Because of these properties, new therapeutic approaches have been developed [57,58], which suggests that the ingestion of fruits with a high content of anthocyanins, among which black fruits stand out, could be recommended.

### 3.4. Total Sugars, Organic Acids and Anthocyanins Content during Long-Term Frozen Storage

The demand for frozen fruits has increased for the last few years and frozen storage could be a better alternative to maintain the quality of the fruits compared to other postharvest treatments [59]. Results from different studies indicate that changes in the contents of polyphenols, nutrients, sugars, and acids during storage and temperature depend on the berry type, cultivar, and fruit maturity [60]. To increase our understanding of these variables on nutraceutical preservation, more comprehensive studies are required, which could be used by processors to develop novel procedures.

**Figure 3:**
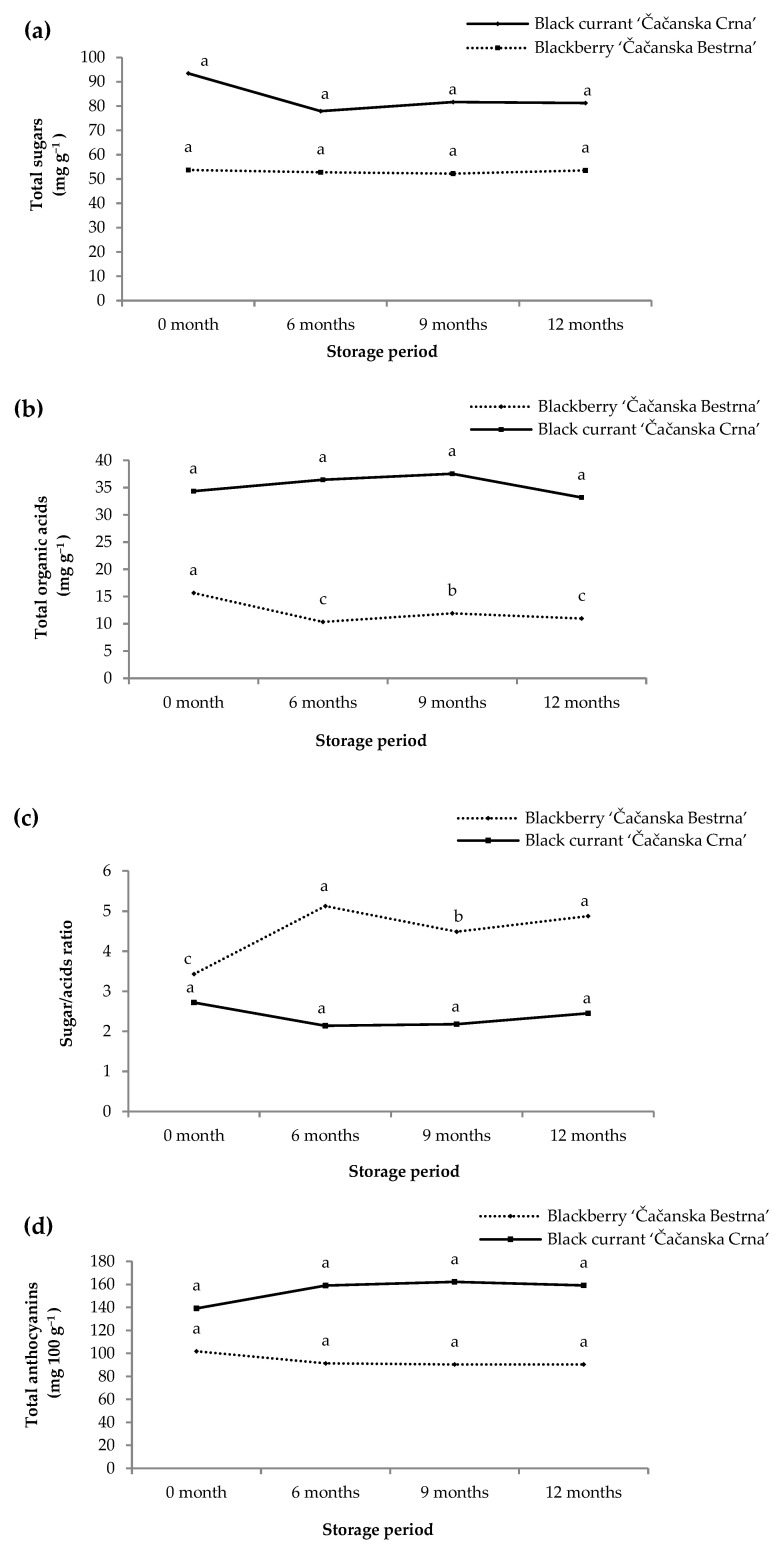
Changes in total sugars (**a**), organic acids (**b**), sugar/acid ratio (**c**), and anthocyanins (**d**) in fruit of blackberry and black currant during freezing and long-term frozen storage (0, 6, 9, and 12 months); Values are mean ± standard deviation (n = 4); Values within each line followed by the same small letter are not significantly different at *p* ≤ 0.05.

Our study determined the effect of long-term frozen storage on the content of nutritional and sensory properties of blackberry and black currant fruits after 6, 9, and 12 months of frozen storage (Figure 3). No frozen storage duration significantly affected the total sugar (TS) values of black currants and blackberry fruit (Figure 3a), pointing towards the stability of the sugar content. Interestingly, Veberic et al. [36] studied the effect of slow and fast freezing of blackberries followed by frozen storage of fruit for 7 months and determined that the highest decrease in sugars content in blackberry ‘Čačanska Bestrna’ was in a slow freezing method (−20 °C) compared to fresh fruits and fast freezing method (liquid N_2_).

Regarding total organic acid (TA) values in blackberry and black currant fruits, a significant effect of storage duration was determined only in blackberry (Figure 3b). The highest TA in blackberry was recorded in the fruits at the beginning of frozen period (15.65 mg g^−1^) and decreased significantly during the storage period. The decrease in TA may be due to acidic hydrolysis of polysaccharides, where acids are used to convert non-reducing sugar to reducing sugar in the fruits [61]. In black currant fruits, TA did not vary significantly during frozen storage. Moreover, Dawson et al. [62] indicates that acids in berries and citric fruits are more stable in frozen fruits compared to other species such as peaches.

Consequently, despite constant sugar content in blackberry samples during frozen storage, sugar/acid (S/A) ratio increased after the sixth month and then decreased moderately (Figure 3c). The results are in accordance with Veberič et al. [36], who indicated that blackberry fruits preserve their characteristic sour-sweet taste even after long-term frozen storage regardless of the initial freezing method or conditions. On the other hand, S/A ratio did not change in black currant samples during frozen storage.

The stability of anthocyanins during freezing and long-term frozen storage depends on the seasonal harvest period, cultivar, and storage duration [36]. In our study, levels of total anthocyanins in blackberry and black currant did not vary after freezing and during frozen storage (Figure 3d), which is consistent with the report of Veberic et al. [36]. Correspondingly, Hager et al. [63] detected that quick freezing and frozen storage of intact blackberry fruits did not result in a loss of total analyzed anthocyanins or their polymerization. The values of anthocyanins in our study were similar to previously reported data on black currant by Oancea et al. [64], and their research reported stability of anthocyanins after 6 months of frozen storage, as well as an increase in 11% in wild and 52% in cultivated black currant after 7 months of frozen storage. Furthermore, Djordjević et al. [10] measured 15% more total anthocyanins in the red currant cultivar ‘Rondom’ after 1 year of freezing. Our results for black currant revealed that total anthocyanins increased after 6 (12%) and 9 months (16%) and then remained constant until 12 months of storage. Accumulation of total anthocyanins during prolonged frozen storage can be explained by fruit cell destruction during freezing and ice sublimation which contributes to a higher extraction efficiency [65].

## 4. Conclusions

Valuable insights into the yield and fruit quality parameters of the black fruits of blackberry cultivar ‘Čačanska Bestrna’ and black currant cultivar ‘Čačanska Crna’, as well as the variations in fruit quality during frozen storage was provided. High content of phenolics, balanced sugar/organic acids ratio, and high productivity observed in ‘Čačanska Bestrna’ suggest that this cultivar is well-suited for expanding plantations. Similarly, the black currant cultivar ‘Čačanska Crna’ with its high values of soluble solids, sweetness index, and anthocyanins can serve as a valuable source of valuable compounds. Different durations of frozen storage did not significantly affect the sugar content in black currants and blackberries, as both species maintained stable levels throughout storage. However, the total acidity of blackberries increased during storage, while in black currants it remained constant. Additionally, total anthocyanins remained constant in both blackberry and black currant fruits during frozen storage. Overall, our results suggest that frozen storage can be a viable option for preserving the quality of blackberry and black currant fruits while retaining their beneficial properties. Additionally, the high phytochemical level of both cultivars highlights the potential for their use in berry breeding programs to create cultivars with improved nutritional value.

## Figures and Tables

**Table 1:** Tested cultivars and their characteristics.

	Blackberry ‘Čačanska Bestrna’	Black Currant ‘Čačanska Crna’
Origin	‘Dirksen Thornless’ × ‘Black Satin’	Open fertilization of ‘Malling Jet’
Harvest time	Mid early (the second ten-day period of July to the third ten-day period of August)	Mid early (during the second and third ten-day period of June)
Fruit size	Large	Medium
Purpose	Combined traits (fresh consumption, freezing, and processing)	

**Table 2:** Fruit weight, soluble solids content, and yield of blackberry and black currant.

Parameters	Blackberry ‘Čačanska Bestrna’	Black Currant ‘Čačanska Crna’	
Fruit weight (g)	8.20 ± 1.29	1.05 ± 0.02	*
Soluble solids content (°Bx)	9.40 ± 1.19	15.23 ± 0.13	*
Yield per bush (kg)	8.01 ± 0.12	3.50 ± 0.35	*
Yield per unit area (t ha^−1^)	17.80 ± 0.26	7.78 ± 0.77	*

Values are mean ± standard deviation (n = 4). (*) indicate significant differences between cultivars (Student’s *t*-test, *p* < 0.05).

**Table 3:** Content of individual and total sugars and organic acids (mg g^−^^1^), sugar/acid ratio, and sweetness index of blackberry and black currant.

Compound	‘Čačanska Bestrna’ Blackberry	‘Čačanska Crna’ Black Currant	
Fructose	28.65 ± 8.80	49.95 ± 1.32	*
Glucose	24.01 ± 7.49	38.86 ± 1.19	*
Sucrose	1.05 ± 0.56	4.25 ± 1.46	*
Total sugars	53.71 ± 16.82	93.06 ± 3.17	*
Citric acid	6.39 ± 0.75	24.16 ± 1.10	*
Malic acid	3.00 ± 0.27	3.68 ± 0.21	*
Quinic acid	5.14 ± 0.33	6.26 ± 0.62	*
Shikimic acid	0.03 ± 0.00	0.24 ± 0.01	*
Fumaric acid	0.01 ± 0.00	0.01 ± 0.00	*
Total organic acids	15.75 ± 0.83	34.35 ± 1.58	*
Sugars/acid ratio	3.39 ± 0.95	2.77 ± 0.10	ns
Sweetness index	91.32 ± 28.44	159.48 ± 4.79	*
Maturity index	0.60 ± 0.17	0.44 ± 0.02	ns

Values are mean ± standard deviation (n = 4). (*) and ns indicate significant and not significant, respectively (Student’s *t*-test, *p* < 0.05).

**Table 4:** Identification and quantification of individual phenolic compounds (mg 100 g^−^^1^) in blackberry and black currant.

Compounds	Blackberry ‘Čačanska Bestrna’	Blackcurrant ‘Čačanska Crna’	
** I Phenolic acids**			
** Hydroxycinnamic acids**			
1. 3-*p*-coumaroylquinic acid	0.06 ± 0.02	n.d.	
2. neochlorogenic acid	59.28 ± 23.81	n.d.	
3. caffeic acid hexoside 1	1.48 ± 0.60	6.21 ± 3.08	
4. caffeic acid hexoside 2	3.30 ± 1.37	n.d.	
5. *p*-coumaric acid hexoside 1	0.92 ± 0.37	n.d.	
6. *p*-coumaric acid hexoside 2	0.10 ± 0.04	n.d.	
7. cryptochlorogenic acid	0.81 ± 0.26	n.d.	
8. ferulic acid hexoside	n.d.	0.27 ± 0.18	
** Hydroxybenzoic acids**			
9. ellagic acid pentoside 1	0.17 ± 0.10	n.d.	
10. ellagic acid pentoside 2	0.14 ± 0.04	n.d.	
11. methylellagic acid pentoside 1	0.50 ± 0.06	n.d.	
12. methylellagic acid pentoside 2	0.09 ± 0.06	n.d.	
13. ellagic acid-deoxyhexoside	n.d.	10.62 ± 4.64	
**Total phenolic acids**	**66.85 ± 2.68**	**17.10 ± 1.44**	*
** II Flavanols**			
14. catechin	2.91 ± 3.26	n.d.	
15. epicatechin	2.72 ± 2.14	n.d.	
16. procyanidin dimer 1	26.52 ± 10.98	n.d.	
17. procyanidin trimer 1	6.85 ± 5.44	n.d.	
18. procyanidin trimer 2	7.05 ± 2.77	n.d.	
19. procyanidin trimer 3	7.94 ± 3.23	n.d.	
**Total flavanols**	**53.99 ± 3.52**	n.d.	
** III Flavonols**			
20. quercetin-3-rutinoside	0.20 ± 0.10	2.05 ± 0.84	*
21. isorhamnetin-3-glucuronide	0.21 ± 0.11	n.d.	
22. quercetin-3-galactoside	0.61 ± 0.30	0.23 ± 0.07	*
23. quercetin-3-glucoside	0.23 ± 0.18	0.61 ± 0.28	ns
24. quercetin-3-xyloside	0.06 ± 0.02	n.d.	
25.quercetin-3-(6″-(hydroxyl-3-methylglutaroyl)-hexoside	1.47 ± 0.62	n.d.	
26. quercetin-3-malonyl-glucoside	n.d.	0.17 ± 0.06	
27. quercetin-3-glucuronide	0.24 ± 0.23	n.d.	
28. quercetin-3-arabinopyranoside	0.02 ± 0.01	n.d.	
29. kaempferol hexoside	0.06 ± 0.05	n.d.	
30. kaempferol-3-glucoside	n.d.	0.53 ± 0.23	
31. kaempferol-3-rutinoside	n.d.	0.09 ± 0.04	
32. quercetin-3-arabinofuranoside	0.20 ± 0.17	n.d.	
33. quercetin-acetylhexoside	0.64 ± 0.54	n.d.	
34. myricetin-3-rhamnoside	n.d.	1.88 ± 0.81	
35. myricetin-3-glucoside	n.d.	2.12 ± 1.01	
36. myricetin-3-rutinoside	n.d.	0.89 ± 0.42	
** Total flavonols**	**3.94 ± 0.18**	**8.57 ± 0.40**	*
**IV Anthocyanins**			
37. cyanidin-3-rutinoside	4.89 ± 1.71	23.50 ± 12.09	*
38. cyanidin-3-glucoside	81.50 ± 28.46	14.19 ± 7.24	*
39. cyanidin-3-arabinoside	0.49 ± 0.19	n.d.	
40. pelargonidin-3-glucoside	3.18 ± 1.42	n.d.	
41. pelargonidin-3-rutinoside	0.23 ± 0.10	n.d.	
42. cyanidin-3-xyloside	2.20 ± 2.79	n.d.	
43. cyanidin-3-(6″-malonylglucoside)	1.64 ± 0.68	n.d.	
44. cyanidin-3-(6″-dioxalylglucoside)	7.80 ± 3.25	n.d.	
45. delphinidin-3-glucoside	n.d.	38.33 ± 24.72	
46. delphinidin-3-rutinoside	n.d.	59.17 ± 28.30	
47. petunidin-3-rutinoside	n.d.	3.92 ± 2.10	
**Total anthocyanins**	**101.93 ± 73.96**	**139.11 ± 32.93**	ns
**TOTAL PHENOLICS**	**226.71 ± 20.09**	**164.78 ± 54.93**	*

Values are mean ± standard deviation (n = 4). * and ns mean significant and not significant, respectively (Student’s *t*-test, *p* < 0.05); n.d.—not detected.

## Data Availability

The data used to support the findings of this study can be made available by the corresponding author upon request.
